# Hemostasis after percutaneous transfemoral access: A protocol for systematic review

**DOI:** 10.1097/MD.0000000000023731

**Published:** 2020-12-24

**Authors:** Rejane Reich, Lucas Helal, Vanessa Monteiro Mantovani, Eneida Rejane Rabelo-Silva

**Affiliations:** a Graduate Program in Nursing, Universidade Federal do Rio Grande do Sul; b Hospital de Clínicas de Porto Alegre; c Graduate Program in Cardiology and Cardiovascular Sciences, Universidade Federal do Rio Grande do Sul, Porto Alegre; d Universidade Federal do Extremo Sul Catarinense, Criciúma; e Graduate Program in Nursing and Graduate Program in Cardiology and Cardiovascular Sciences, Universidade Federal do Rio Grande do Sul, Porto Alegre, Brazil.

**Keywords:** randomized controlled trials, systematic review, transfemoral access

## Abstract

**Background:**

Access site hemostasis after percutaneous procedures done in the catheterization laboratory still needs to be better studied in relation to such aspects as the different results achieved with different hemostasis strategies, the impact of different introducer sheath sizes, and arterial versus venous access. The objective of this review is to synthesize the available scientific evidence regarding different techniques for hemostasis of femoral access sites after percutaneous diagnostic and therapeutic procedures.

**Methods:**

This review is being reported in accordance with the Preferred Reporting Items for Systematic Review and Meta-Analysis Protocols (PRISMA-P). The primary outcomes will include the following vascular complications: hematoma, pseudoaneurysm, bleeding, minor, and major vascular complications. The secondary outcomes will include the following: time to hemostasis, repetition of manual compression, and device failure. A structured strategy will be used to search the PubMed/ MEDLINE, Embase, CINAHL, and CENTRAL databases. In addition, a handsearch of the reference lists of selected studies will be conducted. The ERIC research database will be queried for the gray literature and ClinicalTrials.gov, for potential results not yet published in indexed journals. Two reviewers will independently screen citations and abstracts, identify full-text articles for inclusion, extract data, and appraise the quality and risk of bias of included studies. If possible, a meta-analysis will be carried out. All estimations will be made using Review Manager 5.3. Statistical heterogeneity will be assessed by considering the *I*^2^ proxy, accompanied with qualitative indicators such as differences in procedures, interventions, and outcomes among the studies. If synthesis proves inappropriate, a narrative review will be undertaken.

**Results:**

This protocol adheres to the PRISMA-P guideline to ensure clarity and completeness of reporting at all phases of the systematic review.

**Conclusion:**

This study will provide synthesized information on different methods used to achieve hemostasis after femoral access.

**Ethics and dissemination:**

Ethical approval number CAAE 19713219700005327. The results of the systematic review will be disseminated via publication in a peer-reviewed journal and through conference presentations.

**Systematic review registration:**

PROSPERO CRD42019140794.

## 1 Introduction

The popularity of endovascular procedures performed via the percutaneous approach has grown exponentially in recent years. This route is now firmly consolidated for diagnostic use,^[1,2]^ and therapeutic procedures are establishing themselves as promising treatment options^[3–5]^ and first-line therapies for patients at high surgical risk.^[6,7]^ Due to their less invasive nature, percutaneous approaches shorten procedure time and hospital stay while reducing complication rates.^[8]^

Since the description of the Seldinger technique,^[9]^ percutaneous transfemoral access has become a cornerstone approach for diagnostic procedures and vascular interventions.^[10]^ It offers ease of access and the possibility of being used for cardiac and extracardiac procedures, as the femorals are large-caliber vessels with little anatomical variation,^[11]^ capable of accommodating introducers and delivery systems for large-caliber devices.^[12–14]^

Once the procedure is complete, the orifice resulting from the puncture must be closed and the bleeding stopped (hemostasis). Despite continuous advances and improvements in equipment and materials and in contemporary femoral access techniques,^[11]^ percutaneous procedures still pose important challenges. Vascular complications, including those related to the site of access, are still a cause of morbidity and are associated with adverse outcomes.^[15]^ In large-diameter transcatheter procedures, hemorrhagic and vascular complications have resulted in increased mortality, length of hospital stay, and health expenditure.^[16]^

Due to the diversity of percutaneous procedures using the femoral approach, especially with different calibers of introducers, there is still no conclusive evidence on the safety of hemostasis with the different methods available. Access-related complications and the effectiveness of closure devices are still a matter of open debate. In a review comparing the use of devices and manual compression, the overall complication rates were similar (12.2% for devices vs 13.1% for manual compression), but varied among different devices on the market.^[17]^

A Cochrane review of 52 studies, which compared devices versus extrinsic compression (sheath ≤9 French), found no difference in the incidence of vascular injury or mortality. When comparing device use and open artery exposure (sheath ≥10 French), no difference was found in the effectiveness or safety of devices with different mechanisms of action.^[18]^

A network meta-analysis demonstrated that the use of vascular closure devices is associated with a reduced risk of hematoma.^[19]^ In another network meta-analysis of closure devices used in femoral access for percutaneous coronary intervention, no device was found to be superior to manual compression in reducing vascular complications. After meta-regression, outcomes were not associated with age, sex, or sheath diameter.^[20]^

With the increasing use of percutaneous procedures, effective hemostasis techniques and early detection and management of vascular complications are essential for high-quality care. Further research is still needed to clarify the role of the different strategies available for achieving access site control and hemostasis after femoral sheath removal, especially those involving the use of vascular closure devices. These studies should contemplate the wide range of procedures performed in the catheterization laboratory, arterial and venous approaches, and the use of different introducer calibers.

## 2 Objectives

To synthesize qualitative (and, if possible, quantitative) evidence on hemostasis after percutaneous transfemoral access for diagnostic and therapeutic procedures. This study was designed with the following research question:

- What is the most effective method to achieve hemostasis after percutaneous transfemoral access?

For greater specificity, the question was later reformulated to:

- Are vascular closure devices effective for achieving hemostasis after percutaneous transfemoral access?

## 3 Methods and analysis

### 3.1 Protocol and registration

This protocol was written following the recommendations of the Preferred Reporting Items for Systematic Review and Meta-Analysis Protocols (PRISMA-P)^[21]^ (see supplementary file 1). This protocol has been registered prospectively in the PROSPERO International Prospective Register of Systematic Reviews^[22]^ (CRD42019140794). Preliminary surveys were carried out in May 2019; the study was entered in July 2019 and PROSPERO registration was granted in October 2019. The database search was revised in March 2020, and the review must be completed in 9 months.

### 3.2 Eligibility criteria

The PICO strategy^[23]^ (Table 1) was used to design the research question and the database search strategies. The study will be restricted to adult patients (age ≥18 years). No restrictions will be placed on sex or gender, race, comorbidities, clinical conditions, or other characteristics, such as elective versus urgent procedure or use of heparin or glycoprotein IIb/IIIa inhibitors during the procedure. There will be no date limit, that is, studies may have been published in any year. We limited our search only to randomized clinical trials, but not of any specific design (e.g., parallel, cross-over, factorial, etc). For feasibility reasons, the search will be limited to reports in English, Spanish, and Portuguese, and for which full-text access is available.

**Table 1:** Description of the PICO (population, intervention, comparator and outcomes) strategy.

Definition	Description
Population	Patients undergoing percutaneous access to a femoral vein or artery, with any caliber introducer, for a diagnostic or therapeutic procedure.
Intervention	Vascular closure device (clip or clamp, suture, collagen, sealant, or gel) on immediate withdrawal of the femoral introducer after the procedure is completed.
Comparator	Vascular closure device (clip or clamp, suture, collagen, sealant, or gel) or extrinsic (manual or mechanical) compression on immediate withdrawal of the introducer or according to the study's routine.
Outcomes	Primary outcomes: vascular complications related to the site of access until hospital discharge: hematoma, pseudoaneurysm, bleeding, minor vascular complication, and major vascular complication. Secondary outcomes: time to hemostasis, repetition of manual compression, and device failure.

All of the outcomes will be treated as defined by the authors. Effect sizes will be preserved in their original unit and presented as stated by the authors.

### 3.3 Information sources and search strategies

A systematic strategy will be used to search the PubMed/Medical Literature Analysis and Retrieval System Online (MEDLINE), Embase, Cumulative Index to Nursing and Allied Health Literature (CINAHL), and CENTRAL databases. In addition, a handsearch of the reference lists of the included studies for overlooked relevant articles (including reviews and meta-analyses published on the subject) will be conducted, and the Education Resources Information Center (ERIC) research database will be queried for the gray literature (e.g., theses, dissertations, conference proceedings). We will also search ClinicalTrials.gov for ongoing or potentially yet-unpublished studies. Search queries will be based on the following terms: hemostatic techniques, vascular closure devices, instrumental seal, manual compression, femoral artery, femoral vein, and femoral access; a filter for randomized clinical trials will be applied. The example search strategy in Table 2 will be used for PubMed. This search strategy will be modified and used for the other databases.

**Table 2:** Search strategy will be used for PubMed.

Search strategy
Search (Hemostatic Techniques[mh] OR Vascular Closure Devices[mh] OR Hemostatic Technique^∗^[tw] OR vascular closure device^∗^[tw] OR arterial closure device^∗^[tw] OR arteriotomy closure[tw] OR hemostasis device^∗^[tw] OR VHD OR Instrumental Seal^∗^[tw] OR vascular Seal^∗^[tw] OR arterial Seal^∗^[tw] OR manual compression^∗^[tw] OR manual pressure[tw]) AND (Femoral Artery[mh] OR Femoral Vein[mh] OR Femoral Arter^∗^[tw] OR Femoral Vein^∗^[tw] OR femoral access^∗^[tw]) AND ((clinical[tw] AND trial[tw]) OR clinical trials as topic[mh] OR clinical trial[pt] OR random^∗^[tw] OR random allocation[mh] OR therapeutic use[mh]

### 3.4 Data extraction

One reviewer (RR) will perform the literature search. The results will be entered into the EndNote (V.X9) reference manager^[24]^;duplicates will be eliminated using the provided software tools, and thereafter manually if needed. The remaining files will be shared with the second reviewer (VMM). Both reviewers will independently screen titles and abstracts for eligibility. Full texts will then be obtained for those that meet the inclusion criteria or are deemed uncertain. The full text will then be screened, and the reviewers will independently decide whether the study meets all the requirements for inclusion. The reviewers will record the reasons for excluding studies. Discrepancies will be resolved by discussion between the 2 reviewers. If they cannot reach a consensus, one of the senior authors (ERRS or LH) will adjudicate. The investigators will not be blind to the journal titles, institutions, or study authors. To avoid double-counting, when there are multiple studies from the same cohort, the study with the largest sample size will be used. The selection process will be displayed in a PRISMA flow diagram.^[25]^

Both reviewers will independently use a standardized Excel spreadsheet to extract and collate data of interest from the studies included in the review. The 2 spreadsheets will be compared, and the reviewers will discuss the differences until the worksheets contain the same information. If any difficulties arise in this process, one of the senior authors (ERRS or LH) will be contacted to address the issue.

To assess the risk of bias, the modified Cochrane Risk of Bias Tool (RoB) 1.0 as embedded in Review Manager software (RevManV.5.3 Copenhagen: The Nordic Cochrane Centre, The Cochrane Collaboration)^[26]^ will be used. Bias will be assessed through judgment (high, low, or unclear) of individual elements according to predefined domains (selection, performance, detection, attrition, reporting, and other).^[27]^

Data collection will comprise 4 main areas of information:

- Article: author, title, year of publication, and journal.
- Study characteristics: study design, methodological quality items (random sequence generation, allocation concealment, blinding of participants and personnel, blinding of outcome assessment, incomplete outcome data, selective reporting, and other bias, bias not covered in the other domains of the tool), number of patients, mean patient age, purpose of the procedure (diagnostic or therapeutic intervention; also specifying the procedure), type of access (arterial or venous), caliber of the introducer, procedures to promote hemostasis (vascular closure device—clip or clamp, suture, collagen, sealant and gel, or extrinsic compression—manual or mechanical).
- Results: number of patients per study group, number of patients developing hematomas, number of patients with pseudoaneurysms, number of patients with bleeding, number of patients with minor vascular complication, number of patients with major vascular complication, time to hemostasis, number of patients requiring repetition of manual compression, number of patients with device failure.
- Notes: language of the study and any other information relevant to this review.

### 3.5 Data synthesis

In this review, results will be presented and synthesized using a narrative approach and thematic synthesis, which will be structured according to the central theme. To support the narrative overview, we will create tables summarizing the data extracted in the collection process. Analysis categories will be structured on the basis of hemostasis methods and outcomes.

If data are pertinent for quantitative analysis (i.e., at least 2 studies), we will use a meta-analytical approach. All statistical analyses will be performed using RevMan software^[26]^ and the results of the data analysis will be shown in a forest plot. For continuous data, summary effect sizes will be estimated by gross weighted mean differences and 95% confidence intervals (CIs) under the random effects model and the inverse of variance test. For categorical variables, relative risks and 95% CIs through a DerSimonian-Laird test and random effects model will be used. The inconsistency test (*I*^2^) will be used to assess the heterogeneity of the studies. We will adopt a threshold of 50% to consider a summary estimate with low heterogeneity. If relevant, subgroup analyses will be performed to assess possible causes of heterogeneity. Planned subgroup analyses include those by type of procedure (diagnosis or intervention), type of access (arterial or venous), and introducer caliber (≤6 or >6 French). Finally, the asymmetry of evidence will be analyzed through a funnel plot, which, together with Egger test, will be used to estimate the potential for publication bias.

### 3.6 Certainty of the evidence

The certainty of the evidence will be classified according to the Grading of Recommendations Assessment, Development and Evaluation (GRADE)^[28]^ method. In this system, the certainty of evidence can be scored as high (very confident that the true effect lies close to the estimated effect), moderate (moderately confident in the estimated effect: the true effect is likely to be close to the estimated effect, but there is a possibility that it is substantially different), low (confidence in the estimated effect is limited: the true effect may be substantially different from the estimated effect), or very low (very little confidence in the estimated effect: the true effect is likely to be substantially different from the estimated effect). An evidence table will be developed for presenting evidence and the corresponding results.^[29,30]^ The specifications contained in the GRADE handbook will be followed for classification.^[29]^

## 4 Discussion

This comprehensive synthesis will include different procedures carried out in the interventionist scenario using a transfemoral approach, regardless of the introducer caliber used. It will provide synthesized information on different methods used to achieve hemostasis after femoral access, especially the use of vascular closure devices, in which the overall complication rates are similar, but vary between different devices on the market.^[17–20]^ There is still no conclusive evidence in previous reviews of better device effectiveness in hemostasis.

### 4.1 Limitations of this study

- Anticipated potential limitations include a high degree of heterogeneity.
- The restriction on language of publication may prevent relevant research from being identified and included.
- The decision not to gather data from industry sources may lead to publication bias due to hidden/unpublished data or negative results.

## 5 Conclusion

We will base our conclusions only on findings from the quantitative or narrative synthesis of included studies for this review.

## Acknowledgments

The authors would like to thank the reference librarians at the Universidade Federal do Rio Grande do Sul Medical School Library for their assistance with the search queries.

## Author contributions

**Conceptualization:** Rejane Reich, Lucas Helal, Eneida Rejane Rabelo-Silva.

**Investigation:** Rejane Reich, Vanessa Monteiro Mantovani.

**Methodology:** Rejane Reich, Lucas Helal, Eneida Rejane Rabelo-Silva.

**Software:** Rejane Reich, Lucas Helal, Eneida Rejane Rabelo-Silva.

**Supervision:** Lucas Helal, Eneida Rejane Rabelo-Silva.

**Validation:** Rejane Reich, Lucas Helal, Vanessa Monteiro Mantovani, Eneida Rejane Rabelo-Silva.

**Writing – original draft:** Rejane Reich, Lucas Helal, Eneida Rejane Rabelo-Silva.

**Writing – review & editing:** Rejane Reich, Lucas Helal, Vanessa Monteiro Mantovani, Eneida Rejane Rabelo-Silva.

## Supplementary Material

Supplemental Digital Content
